# HIV patients in the ICU: our experience

**DOI:** 10.1186/cc11006

**Published:** 2012-03-20

**Authors:** V Nunes Velloso, L Calejman, E Canedo, M Deheza

**Affiliations:** 1 Hospital Rivadavia, Capital Federal, Argentina

## Introduction

The objective was to describe characteristics of HIV-positive patients admitted to the ICU.

## Methods

HIV-positive patients admitted between February 2000 and February 2011, and demographic data, APACHE II score, cause of admission, days of internment, need for mechanical ventilation (MV), previous antiretroviral therapy of high efficacy before admission (HAART), viral load and CD4 count.

## Results

A total of 3,568 patients were admitted; 715 patients (20.03%) were HIV-positive, 413 patients (57.76%) were masculine and 302 patients (42.23%) feminine, and average age was 33 for men and 35 for women. The APACHE II average score was 13 versus 15.28 for the general population. The most frequent cause of admission was respiratory failure in 329 patients (46%), 57% due to *Pneumocystis jivoreci *and bacterial pneumonias in 35%, the most frequent bacteria isolated were Streptococcus, *Staphylococcus aureus *and *Haemophilus influenzae*. There were two cases of respiratory Kaposi sarcoma and 26 cases of *Mycobacterium tuberculosis*. Other causes were decrease in mental state in 157 patients (22%), with the most frequent causes reported being toxoplasmosis, cryptococcus neoformans and brain lymphoma, immediately post surgery in 79 patients (11%), COPD reagudization and asthma (9%), digestive bleeding in 36 patients (5%) and renal insufficiency in 50 patients (7%). From the 715 HIV-positive patients admitted, 479 required MV (67%). Regarding nationality, 276 (38.6%) patients were Argentinean, and the other nationalities were Bolivian, Paraguayan, Peruvian and Korean. The average length of stay was 10.5 days and the mortality was 43%. The viral load average was inferior to 10^4 ^RNA/ml in just 44 known patients and the CD4 count was determined in 75 patients, from which the average was 400/mm^3^. The proportion of patients receiving HAART was just 26%.

## Conclusion

HIV-positive patients have a high frequency of admission to the ICU, and they have a lower risk score in comparison with non-HIV patients. The two main causes of admission where respiratory disease and infectious CNS disease. Significant results were the prevalence of patients from limited countries, high mortality and prolonged stay in the ICU, and poor adherence to antiretroviral therapy.

